# Acute transverse myelitis (ATM) associated with COVID 19 infection and vaccination: A case report and literature review

**DOI:** 10.3934/Neuroscience.2024011

**Published:** 2024-06-11

**Authors:** Srinivas Medavarapu, Nitasha Goyal, Yaacov Anziska

**Affiliations:** 1 Department of Neurocritical care, Mount Sinai and and Department of Neurology, State University of New York Downstate, Brooklyn, NY, USA; 2 State University of New York Downstate Medical School, Brooklyn, NY, USA; 3 Department of Neurology, Neuromuscular Disease, State University of New York Downstate, Brooklyn, NY, USA

**Keywords:** COVID-19, Transverse Myelitis

## Abstract

Acute transverse myelitis (ATM) is an inflammatory disorder caused by many etiologies, from postinfectious to autoimmune. Rarely, ATM cases have been reported after both COVID-19 infection and COVID-19 vaccination. We described our experience with ATM after COVID-19 infection and conducted a literature review.

**Case finding Methods:**

We reported a case of longitudinally extensive ATM after COVID 19 infection, who also received convalescent plasma therapy, and present a comprehensive literature review of ATM cases reported after COVID-19 infection and COVID-19 vaccination. The literature search was done using PubMed and Google scholar with keywords and selected peer-reviewed articles. The search included all cases from Jan 2020 to Sept 2022.

**Results:**

A total of 60 ATM cases reported association with post COVID 19 infection, and 23 ATM cases reported association with post COVID 19 vaccinations. Among post COVID 19 ATM cases, the mean age was 49 years and the youngest reported was 7-month-old. A total of 55% (33) were longitudinally extensive ATM. The most common symptom was lower extremity weakness. One case was reported as necrotizing myelitis on biopsy, and another case overlapped with syndrome of GBS and longitudinal ATM. No cases reported using convalescent plasma therapy after infection. Almost all the ATM cases were treated with steroids, but some cases needed additional treatment since not all responded adequately. Six cases (10%) responded with steroids plus plasmapheresis, and 5 cases (8%) responded with steroids + IVIG, especially in the pediatric age group. One case reported a positive response after treatment with eculizumab, and another with infliximab. Two cases (3%) remained paraparetic. Among post covid-19 vaccine ATM cases, 4 cases (17%) were reported as longitudinally extensive ATM. Five cases (21%) had symptom onset within a week after vaccination. Almost all reported a response to steroids except for one case which reported fatality after the 58^th^ day after vaccination.

**Conclusion:**

ATM, in the setting of acute COVID-19 infection, has been described in multiple cases and is a rare complication of COVID-19 vaccination.

## 1. Background and purpose

Acute transverse myelitis (ATM) is a rare inflammatory disorder, caused by many etiologies, from postinfectious to autoimmune. Only a few ATM cases have been reported after COVID-19 infection and vaccination. We report a case of longitudinally extensive ATM after COVID-19 infection who also received convalescent plasma therapy and present a comprehensive literature review of ATM cases reported after COVID-19 infection and COVID-19 vaccination.

## 2. Introduction

Acute transverse myelitis is an acquired inflammatory disorder that affects approximately 1 to 8 people per 1 million each year [1]. It is commonly idiopathic but can also be caused by a postinfectious complication that is thought to be the result of an autoimmune reaction. Inflammatory transverse myelitis usually manifests as weakness, sensory impairments, or bladder and bowel dysfunction. It is a clinical condition caused by immune-mediated spinal cord injury characterized by sensory, motor, and autonomic dysfunction. Common infectious causes are enteroviruses, West Nile virus, herpes, and HIV [1]. Cases of COVID-19 being an infectious cause of ATM have been reported in both underdeveloped and developed countries [2].

Since its onset, the gravity of COVID-19 can be seen worldwide with 767,750,853 confirmed cases including 6,941,095 deaths [3]. Infection with COVID-19 causes an inflammatory response associated with reports of coughing, myalgias, headaches, diarrhea, sore throats, and abnormal tastes or smells [4]. Pneumonia is one of the most common serious manifestations of the infection but there are also associations with a number of neurological conditions such as acute cerebrovascular disease and encephalopathy, autoantibody-mediated diseases such as Guillain-Barré syndrome, and inflammatory complications such as transverse myelitis [5]–[9]. Vaccinations for Covid-19 became available in late 2020 and since then numerous cases of post-vaccination neurological disorders have been reported [10],[11]. In recent years, Pfizer-BioNTech, Moderna, and Johnson & Johnson's Covid-19 vaccines have been associated with a few cases of acute ATM. These findings suggest a potential association between COVID-19 and ATM which warrants further investigation. Here we present a young female with symptoms of ATM 12 days after COVID 19 infection treated with convalescent plasma therapy.

## 3. Case description

A 63-year-old Hispanic woman with a past medical history of hypertension presented with 4 days of lower back pain, numbness/burning sensations from the umbilicus into both legs, paraplegia, bowel and bladder incontinence, and saddle anesthesia. Her symptoms initially started in her legs with numbness that gradually progressed to weakness. Approximately 12 days prior to the onset of her symptoms, she experienced fever, chills, and shortness of breath and was diagnosed with severe acute respiratory syndrome coronavirus 2 (SARS-CoV-2). She was admitted to the hospital for one day, received convalescent plasma therapy and was discharged with prophylactic anticoagulation (Apixaban 5mg BID). After discharge, the patient returned to her usual state of health until she started experiencing these new symptoms.

**Figure 1.**
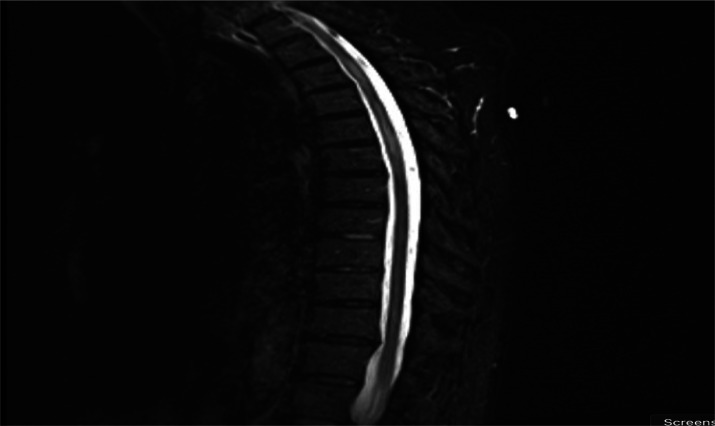
MRI of the thoracic spine in saggital view - T4-7 non-enhancing intramedullary lesion suggestive of transverse myelitis.

**Figure 2.**
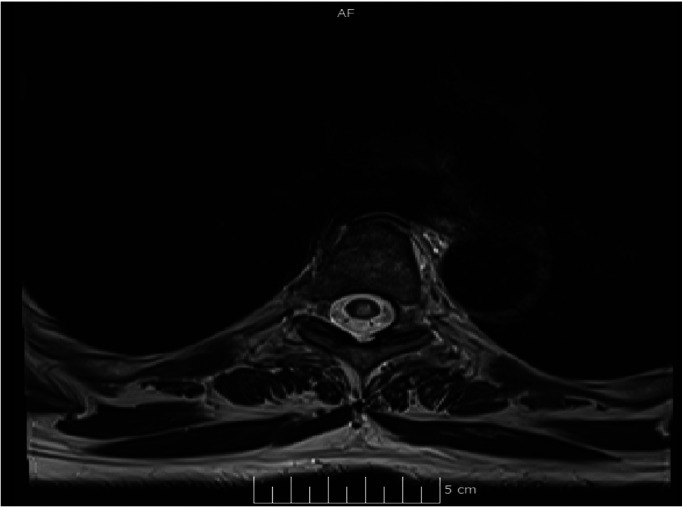
MRI of the thoracic spine in axial view with a non-enhancing intramedullary lesion suggestive of transverse myelitis.

Upon physical examination, the patient was afebrile, normotensive, and breathing comfortably. Her heart sounds were regular with a sinus rhythm, the lungs were clear to auscultation, and the abdomen soft. The neurological exam demonstrated normal mental status, including language and attention, intact cranial nerves, a T10 sensory level defect, mild left hip flexion and knee extension weakness, and hyperreflexia and absent vibration sensation in the bilateral lower extremities. The only lab abnormality was a low-normal B12 of 290 (borderline is 200–300). MRI of the thoracic spine without contrast initially showed an unclear signal change and repeat MRI of the thoracic spine with and without contrast confirmed longitudinal extensive myelitis (LETM), with a T4-7 non-enhancing intramedullary lesion suggestive of transverse myelitis (Figure 1 and Figure 2). CSF studies were unremarkable without pleocytosis or elevated protein. CSF gram stain, culture and serology tests for herpes zoster, varicella-zoster, and Epstein-Barr virus were negative. The serum Aquaporin-4 antibody was negative. Autoimmune immunological screening, including Lupus anticoagulant, Protein S and C levels, Anti-Neutrophil Cytoplasmic antibodies, Rheumatoid factor (RF), Anti Cardiolipin, and Anti-Beta 2 Glycoprotein, were all negative. The patient was subsequently diagnosed with transverse myelitis, and after ruling out infectious causes, she was treated with a 5-day course of parenteral methylprednisolone (1 gram for each session), which resulted in mild improvement of symptoms.

## 4. Discussion

ATM is a disorder of the nervous system characterized by inflammation of the spinal cord without a compressive lesion. Approximately 3 patients per 100,000 patients (0.003%) suffer from acute transverse myelitis [12]. As a result of an immunological reaction, acute transverse myelitis (ATM) presents with sudden-onset motor weakness, sensory loss, and bowel or bladder dysfunction. MRI scans can be used to confirm the diagnosis of acute transverse myelitis (ATM) [13]. The most common causes are multiple sclerosis, neuromyelitis optica spectrum disorder, and active infections but it can also be caused by systemic diseases, paraneoplastic disorders, and post-infectious conditions [14]. More recently, COVID-19 infection and vaccination have been linked as a potential predisposing cause of ATM.

The effects of the COVID 19 epidemic are still being understood. Amongst covid neurological complications, the most frequent ones are cerebrovascular complications, Guillain-Barre, and ATM. According to a retrospective study in China by Mao et al., 214 patients suffered from neurological symptoms associated with COVID-19 [15]. Among the neurological complications associated with COVID-19, ATM was found to be an unexpectedly frequent one. Researchers at Houston Methodist Hospital found 43 cases of acute transverse myelitis (ATM) associated with COVID-19 patients, and an April 2021, researchers found that ATM represents 1.2% of all COVID-19 neurological complications [16]. An additional study examined 43 patients from 21 countries with COVID-19-associated ATM. Approximately 68% of the cases had a latency of 10 days to 6 weeks, which may be indicative of postinfectious neurological complications. In 32% of cases, there was a short latency (15 hours to 5 days) suggesting a direct neurotropic effect of SARS-CoV-2 [16].

There have been reports of not only the infection itself increasing risk of ATM but also the vaccines. The United States has administered 51,755,447 doses of different vaccines as of March 2, 2021. According to the vaccine adverse event reporting system, of the 9,442 adverse reactions associated with Pfizer-BioNTech, Moderna, and Johnson & Johnson's Covid-19 vaccines, 254 were neurological, including 9 cases of ATM [17]. The COVID-19 vaccine produced by Oxford-AstraZeneca uses chimpanzee adenoviral vectors (ChAdOx1) that contain the glycoprotein antigen (spike protein) gene from the SARS-CoV-2. As a result of the Oxford-AstraZeneca COVID-19 vaccine, three cases of ATM have been reported [18]. In the AZD1222 vaccine trial, 3 ATM adverse events were reported among 11,636 participants, which is extremely high at 0.5 cases per million COVID-19-associated ATM cases worldwide [16].

An acute transverse myelitis (ATM) complication of vaccination has been reported by Hyunjong Eom, 2022. They describe two cases of ATM following the administration of an mRNA vaccine for Coronavirus disease 2019 (COVID-19). Both patients received the BNT162b2 vaccine. One of them is an 81-year-old man with weakness in both hands. A high signal intensity was observed between the C1 and C3 vertebrae on a spine magnetic resonance imaging (MRI). In the second case, a 23-year-old woman experienced tingling in her legs. It was found that the conus medullaris had a high signal intensity lesion. During treatment, methylprednisolone was administered intravenously for five days, followed by oral prednisolone taper for two weeks and two months. These findings suggest the need for careful follow up after the administration of mRNA vaccines to detect potential adverse events [19].

According to the Transverse Myelitis Consortium Working Group, another study revealed a patient with longitudinally extensive transverse myelitis. This patient's ATM developed 3 weeks after vaccination, and after excluding all other possible etiologies, the timing between vaccination and onset of symptoms was consistent with post vaccination myelitis. Covid-19 was not detected in the patient, ruling out postinfectious myelitis. There was no evidence of intracranial lesions in brain imaging or OCB, NMOSD (Neuro Myelitis Optica Spectrum Disorder) and MOG antibodies (Myelin oligodendrocyte glycoprotein) in CSF examination; therefore, it was unlikely that the patient had multiple sclerosis (MS), NMO spectrum disorders, or MOG-associated disorders. ATM post-vaccination was the only suspect after vasculitis and paraneoplastic screenings were unremarkable [14].

**Figure 3.**
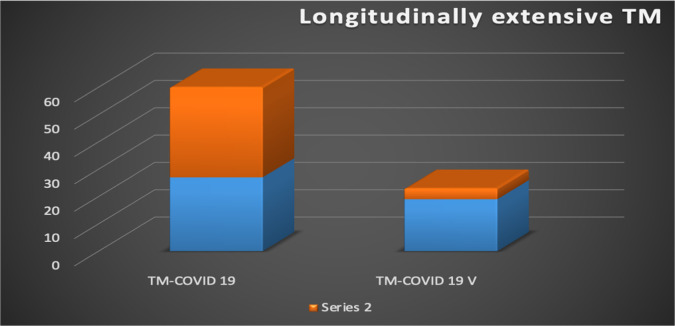
Among post COVID 19 ATM cases 55% were longitudinally extensive ATM and among post covid-19 vaccine ATM cases 17% were reported as longitudinally extensive ATM.

Combined a total of 60 ATM cases reported association with post-COVID-19 infection [16],[20]–[35] and 23 ATM cases reported association with post-COVID-19 vaccinations [17],[36]–[49]. Among post-covid-19 vaccine ATM cases, 4 cases (17%) were reported as longitudinally extensive ATM (Figure 3). Five cases (21%) had symptom onset within a week after vaccination. Among post-COVID-19 ATM cases, the mean age was 49 years, and the youngest reported was 7 months old. A total of 55% (33) of them are longitudinally extensive ATM. The most common symptom is lower extremity weakness. One case was reported as necrotizing myelitis on biopsy [22], and another case overlapped with syndrome of GBS with longitudinal ATM [26]. Almost all ATM cases were treated with steroids, but some cases needed additional treatment since not all responded adequately. Six cases (10%) responded with steroids plus plasmapheresis, and 5 cases (8%) responded with steroids + IVIG, especially in the pediatric age group. One case reported a positive response after treatment with eculizumab, and another with infliximab. 2 cases (3%) remained paraparesis. Nearly all reported responding with steroids except one case, which reported fatality after the 58th day after vaccination [43]. No cases reported the use of convalescent plasma therapy after infection. In our case, the patient received convalescent plasma therapy for COVID-19 and was recovering when she was diagnosed with transverse myelitis and was treated with a 5-day course of parenteral methylprednisolone (1 gram for each session), which resulted in mild improvement of symptoms.

According to reports, vaccine-related disorders might need to be included in the list of neurological disorders caused by COVID-19. There is no doubt that additional data are needed to make a more accurate assessment of the actual relevance and potential risk. Despite this finding, vaccinations should not be avoided in light of the rarity of this occurrence, as their use is one of the most important strategies for fighting an outbreak.

## 5. Conclusions

Transverse myelitis is a rare disease newly associated with increased risk from COVID-19 infections and vaccinations. ATM, in the setting of acute COVID-19 infection, has been described in multiple cases, and is a rare complication of COVID-19 vaccination.
